# Correlative near-infrared light and cathodoluminescence microscopy using Y_2_O_3_:Ln, Yb (Ln = Tm, Er) nanophosphors for multiscale, multicolour bioimaging

**DOI:** 10.1038/srep25950

**Published:** 2016-05-17

**Authors:** S. Fukushima, T. Furukawa, H. Niioka, M. Ichimiya, T. Sannomiya, N. Tanaka, D. Onoshima, H. Yukawa, Y. Baba, M. Ashida, J. Miyake, T. Araki, M. Hashimoto

**Affiliations:** 1 Graduate School of Engineering Science, Osaka University, 1-3 Machikaneyama-cho, Toyonaka, Osaka 560-8531, Japan; 2 Institute for NanoScience Design, Osaka University, 1-3 Machikaneyama-cho, Toyonaka, Osaka 560-8531, Japan; 3 School of Engineering, The University of Shiga Prefecture, 2500 Hassaka-cho, Hikone, Shiga 522-8533, Japan; 4 Department of Innovative and Engineered Materials, Tokyo Institute of Technology, 4259 Nagatsuta, Yokohama, Kanagawa 226-8503, Japan; 5 Quantitative Biology Center (QBiC), RIKEN, 1-3 Yamadaoka, Suita, Osaka 565-0874, Japan; 6 Institute of Innovation for Future Society, Nagoya University, Furo-cho, Chikusa-ku, Nagoya 464-8603, Japan; 7 ImPACT Research Center for Advanced Nanobiodevices, Furo-cho, Chikusa-ku, Nagoya 464-8603, Japan; 8 Graduate School of Engineering, Nagoya University, Furo-cho, Chikusa-ku, Nagoya 464-8603, Japan; 9 Health Research Institute, National Institute of Advanced Industrial Science and Technology (AIST), 2217-14, Hayashi-cho, Taka matsu 761-0395, Japan

## Abstract

This paper presents a new correlative bioimaging technique using Y_2_O_3_:Tm, Yb and Y_2_O_3_:Er, Yb nanophosphors (NPs) as imaging probes that emit luminescence excited by both near-infrared (NIR) light and an electron beam. Under 980 nm NIR light irradiation, the Y_2_O_3_:Tm, Yb and Y_2_O_3_:Er, Yb NPs emitted NIR luminescence (NIRL) around 810 nm and 1530 nm, respectively, and cathodoluminescence at 455 nm and 660 nm under excitation of accelerated electrons, respectively. Multimodalities of the NPs were confirmed in correlative NIRL/CL imaging and their locations were visualized at the same observation area in both NIRL and CL images. Using CL microscopy, the NPs were visualized at the single-particle level and with multicolour. Multiscale NIRL/CL bioimaging was demonstrated through *in vivo* and *in vitro* NIRL deep-tissue observations, cellular NIRL imaging, and high-spatial resolution CL imaging of the NPs inside cells. The location of a cell sheet transplanted onto the back muscle fascia of a hairy rat was visualized through NIRL imaging of the Y_2_O_3_:Er, Yb NPs. Accurate positions of cells through the thickness (1.5 mm) of a tissue phantom were detected by NIRL from the Y_2_O_3_:Tm, Yb NPs. Further, locations of the two types of NPs inside cells were observed using CL microscopy.

Correlative light–electron microscopy (CLEM) is an emerging technique that combines the advantages of both light and electron microscopic techniques to observe a region of interest^1^,^2^,^3^,^4^,^5^. Light microscopy visualizes cellular components and protein distributions using fluorescent probes of different colours. However, positions of proteins cannot be resolved precisely, because the spatial resolution of light microscopy is limited by the diffraction limit of light. To address this issue, considerable effort has been devoted to the development of super-resolution optical microscopy in the past ten years^6^,^7^,^8^. Nonetheless, electron microscopy still plays an important role in the examination of protein distributions and visualization of ultra-structures of cellular components. Because electron microscopy uses an electron beam, it has a higher spatial resolution compared with conventional light and super-resolution optical microscopies. CLEM overcomes the limitations of the individual microscopic techniques and combines coloured imaging and high-spatial-resolution imaging in the same region of a sample.

We have improved the conventional CLEM technique through various approaches. First, we combined cathodoluminescence (CL) microscopy with CLEM. Cathodoluminescence is induced by accelerated electrons, and the wavelength of emitted photons depends on the imaging probes used^9^,^10^,^11^. CL microscopy has a higher spatial resolution than light microscopy^12^,^13^; thus, it enables high-spatial-resolution coloured imaging of cellular components. Second, we used the near-infrared (NIR) wavelength region for fluorescence imaging. Although conventional fluorescence microscopy has been unable to visualizing deep structures in biological samples, use of highly penetrative NIR light has the advantages of deep tissue observation with lower photo-damage for living organisms *in vivo* and a lower auto-fluorescence background compared with the use of visible light^14^,^15^,^16^.

To achieve correlative CL and NIR light excitation imaging, we have previously investigated the multimodal luminescent properties of rare-earth-doped Y_2_O_3_ nanophosphors (NPs)^17^. Y_2_O_3_:Tm, Yb NPs emit visible light after excitation with both an electron beam and 980 nm NIR light. Thus, colour-based discrimination of CL imaging and deep optical imaging can be achieved in correlative imaging with rare-earth-doped NPs.

The aim of this study is to explore the possibility of correlative bioimaging with NIRL and CL, which to the best of our knowledge has not yet been examined. We developed a new correlative imaging technique that employs NIR excitation–NIR emission and dual-colour CL imaging. Using the NIR region for both excitation and emission light enables deep observation inside the body^18^.

CL imaging allows recognition of the type of target depending on the CL colour. Combining NIR luminescence (NIRL) and CL imaging in the same imaging probes enables realizing a new hybrid imaging technique that has wider applicability than the conventional CLEM technique. We investigated the NIRL and CL properties of rare-earth-codoped NPs, Y_2_O_3_:Tm, Yb and Y_2_O_3_:Er, Yb NPs, with particle sizes of less than 100 nm; using correlative NIRL and CL imaging with these NPs, we observed the identical region of interest in both NIRL and CL images. Furthermore, we conducted multiscale NIRL/CL bioimaging to acquire intracellular images at different scales ranging from the whole-body scale to nanoscale.

## Results

### Evaluation of synthesized Y_2_O_3_ NPs

**Figure 1:**
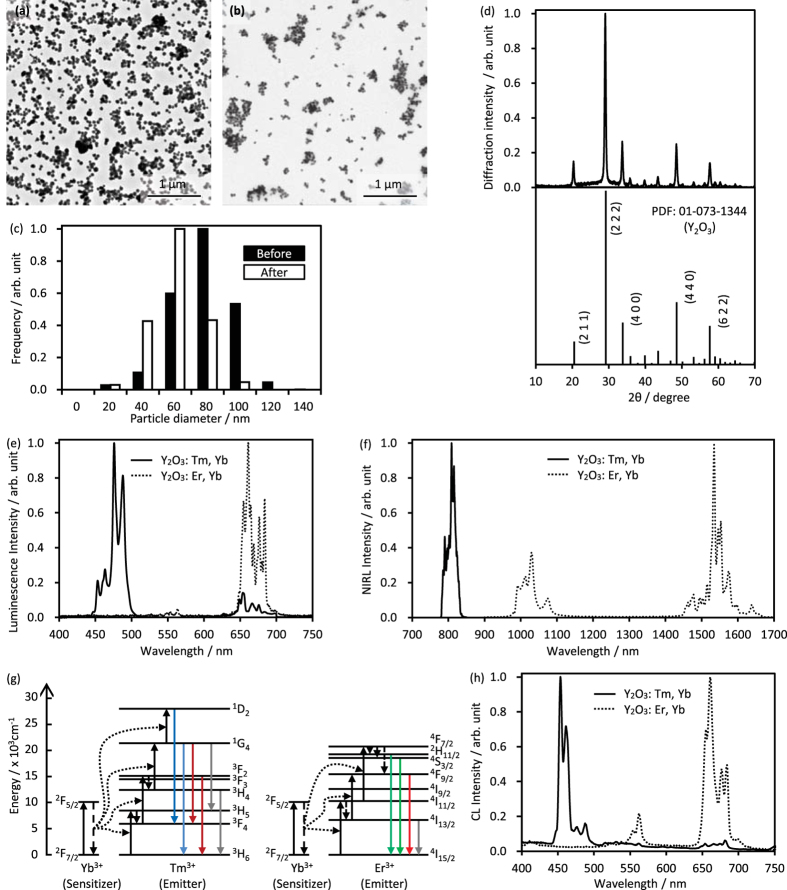
Particle and luminescent properties of Y_2_O_3_ NPs. TEM images of Y_2_O_3_ NPs before (**a**) and after calcination (**b**), and size histogram (**c**) of the NPs. XRD spectrum of the NPs (**d**). Normalized luminescence spectra of Y_2_O_3_:Tm, Yb (solid) and Y_2_O_3_:Er, Yb (dotted) phosphors under 980 nm near-infrared light irradiation (**e,f**). Diagram of energy transfer between sensitizer Yb^3+^ and emitters Tm^3+^ and Er^3+^ (**g**). Normalized cathodoluminescence spectra of Y_2_O_3_:Tm, Yb (solid) and Y_2_O_3_:Er, Yb (dotted) phosphors electron beam irradiation (**h**).

Figure 1(a,b) show TEM images and the size distribution of the Y_2_O_3_:Er, Yb NPs before and after calcination. To obtain small nanoparticles, we increased the nucleation of particles with excess urea, based on our previous studies^11^,^17^. In the homogeneous precipitation method, the particle size depends on the nucleation and particle growth of precursors in the precipitation reaction. Excess nucleation in the initial stage of the precipitation reaction causes a decrease in the rare-earth concentration in solution, consequently suppressing the growth of particles. The precursors had a spherical shape with a size of 69 ± 17 nm. After calcination at 1200 °C, the particle size decreased to 50 ± 14 nm (Fig. 1(c)) because of a morphology change from the rare-earth carbonate hydroxide precursors to rare-earth oxide NPs. Although the NPs exhibited sintering and aggregation after calcination, the aggregates were removed by centrifugation (4 krpm, 10 min), and dispersed NPs were obtained, as shown in Fig. 1(b).

Figure 1(d) shows the XRD spectrum of the Y_2_O_3_:Er, Yb NPs after calcination. The diffraction peaks corresponded to those of cubic Y_2_O_3_, which indicates that precursors were successfully oxidized and crystallized by the calcination process. Analysis of the elemental composition of the NPs using inductively coupled plasma atomic emission spectroscopy (ICP-AES) revealed that Y_2_O_3_:Tm, 0.2; Yb, 2.2 mol% NPs and Y_2_O_3_:Er, 3.2; Yb, 22.0 mol% NPs were obtained.

The luminescence properties of the NPs are shown in Fig. 1(e–h). Under 980 nm NIR light irradiation, the Y_2_O_3_:Tm, Yb and Y_2_O_3_:Er, Yb NPs exhibited luminescence attributed to the *4f-4f* energy transition processes of Tm^3+^ and Er^3+^ activators. The Y_2_O_3_:Tm, Yb NPs exhibited visible luminescence around 490 nm derived from the ^1^G_4 → _^3^H_6_ energy transition of Tm^3+^ and NIRL around 810 nm derived from the ^1^G_4 → _^3^H_5_ and ^3^H_4 → _^3^H_6_ energy transitions of Tm^3+^. The Y_2_O_3_:Er, Yb NPs exhibited visible luminescence at 550 nm and 660 nm derived from the ^2^H_11/2  →  _^4^I_15/2_ and ^4^F_9/2_ → ^4^I_15/2_ energy transitions of Er^3+^, respectively. These emission processes are called as upconversion in which lower energy, longer wavelength excitation light is transduced to higher energy, shorter wavelength emission light. The Y_2_O_3_:Er, Yb NPs exhibited NIRL around 1530 nm derived from ^4^I_13/2 → _^4^I_15/2_ energy transitions of Er^3+ ^19. For the co-dopant Yb^3+^ions, which have a large absorption coefficient of NIR light around 900–1000 nm, the energy of the absorbed NIR light in Yb^3+^ions is transferred to the emitter Tm^3+^ and Er^3+^ ions, which results in multi-photon excitation processes in NPs (Fig. 1(g)). The emission wavelength can be tuned by co-doping with different rare-earth elements, which act as emitter ions. In addition, Y_2_O_3_:Tm, Yb and Y_2_O_3_:Er, Yb NPs exhibited sharp CL peaks at 455, 550, and 660 nm due to the ^1^D_2_ → ^3^F_4_ transition of Tm^3+^, ^4^S_3/2_ → ^4^I_15/2_ transition of Er^3+^, and ^4^F_9/2_ → ^4^I_15/2_ transition of Er^3+^, respectively (Fig. 1(h)). These emission spectra corresponded to those of Y_2_O_3_:Tm and Y_2_O_3_:Er NPs. The results demonstrate that our NPs have multimodal luminescence properties, making them useful for both NIRL and CL microscopy.

### Correlative NIRL/CL imaging of Y_2_O_3_:Tm, Yb and Y_2_O_3_:Er, Yb NPs

**Figure 2:**
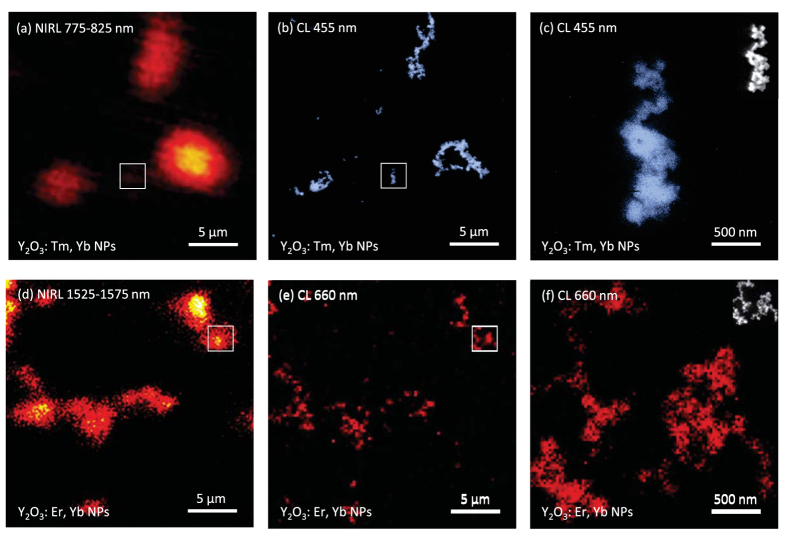
Correlative NIRL (**a,d**) and CL (**b,c,e,f**) images of Y_2_O_3_:Tm, Yb (**a–c**) and Y_2_O_3_: Er, Yb (**d–f**) NPs dispersed on SiN membrane grids. Excitation wavelength: 980 nm (**a,d**). Excitation power: 6.6 mW (**a**) and 7 mW (**d**). Acquisition wavelength: 775–825 nm (**a**), 455 nm (**b,c**), 1525–1575 nm (**d**), and 660 nm (**e,f**). Acceleration voltage of electron beam: 5 kV. Current: 704.5 pA (**b,c**), 724.5 pA (**e**), and 689.5 pA (**f**). Scanning speed: 61.44 μs/pixel (**a,d**), 10 ms/pixel (**b,c,f**), and 2 ms/pixel (**e**).

Figure 2 shows the correlative NIRL and CL images of Y_2_O_3_:Tm, Yb and Y_2_O_3_:Er, Yb NPs, which were dispersed on 50 nm-thick silicon nitride membrane grids and sequentially observed with an NIR microscope and a field emission scanning electron microscopy (FE-SEM)–CL system. Under 980 nm NIR light irradiation, the locations of the Y_2_O_3_:Tm, Yb NPs were observed by utilizing the NIRL emission around 775–825 nm (Fig.2(a)). In contrast, CL images obtained from the same region showed that individual Y_2_O_3_:Tm, Yb NPs emitted blue CL with a peak wavelength of 455 nm under electron beam excitation (Fig. 2(b,c)). The high spatial resolution of CL imaging was confirmed from Fig. 2(c), where the image was acquired at a higher magnification in comparison with those of Fig. 2(a,b). The multimodality of the Y_2_O_3_:Er, Yb NPs was also confirmed in correlative NIRL/CL imaging. Locations of the Y_2_O_3_:Er, Yb NPs were detected with NIRL around 1525–1575 nm, as shown in Fig. 2(d), and CL with a peak wavelength of 660 nm. Owing to the co-dopants Tm^3+^ and Er^3+^, Y_2_O_3_:Tm, Yb and Y_2_O_3_:Er, Yb NPs emit luminescence in different wavelength regions under both 980 nm NIR light and electron beam irradiation. These results demonstrate that the locations of the NPs used as imaging probes can be traced in different-scale imaging utilizing NIRL and CL and the two types of NPs can be differentiated through selection of the luminescent colour.

**Figure 3:**
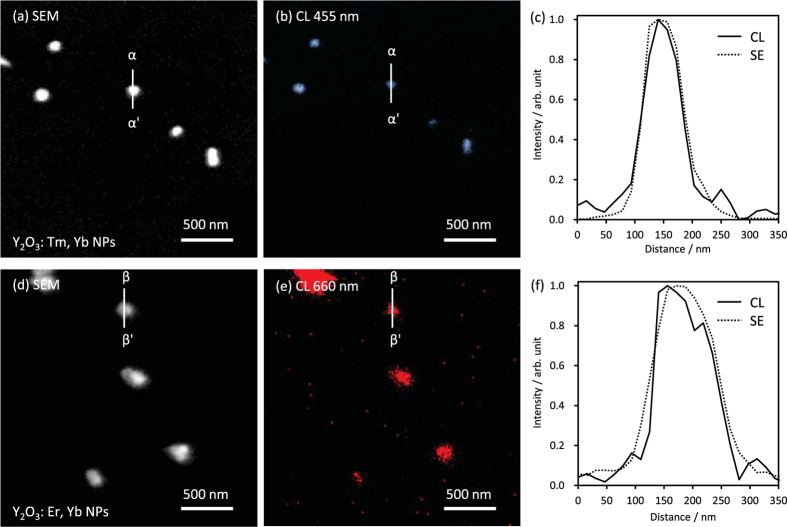
SEM and CL images of Y_2_O_3_:Tm, Yb NPs (**a,b**) and Y_2_O_3_:Er, Yb NPs (**d,e**) on SiN membrane grids. Acquisition wavelength: 455 nm (**b**) and 660 nm (**e**). Acceleration voltage of electron beam: 5 kV. Current: 724.5 pA. Scanning speed: 50 ms/pixel (**b**) and 10 ms/pixel (**e**). Normarized CL (solid) and secondary electron (SE, dotted) intensity profiles along white lines in SEM and CL images (**c,f**).

To investigate the spatial resolution of CL imaging with the NPs, FE-SEM and CL images of the dispersed NPs were obtained as shown in Fig. 3. The Y_2_O_3_:Tm, Yb and Y_2_O_3_:Er, Yb NPs emitted detectable blue or red CL and the location of the CL spots corresponded to the location of NPs in the FE-SEM images. Figure 3(e,f) show the intensity line profiles of the NPs in the FE-SEM and CL images, demonstrating that the CL profiles are consistent with the secondary electron profiles. These results confirm that the spatial resolution of CL imaging reaches that of FE-SEM imaging and the types of NPs are recognized at the single-particle level and with multicolour.

### *In vivo* and *in vitro* deep tissue observation with NIR excitation–NIR emission of Y_2_O_3_:Er, Yb NPs

**Figure 4:**
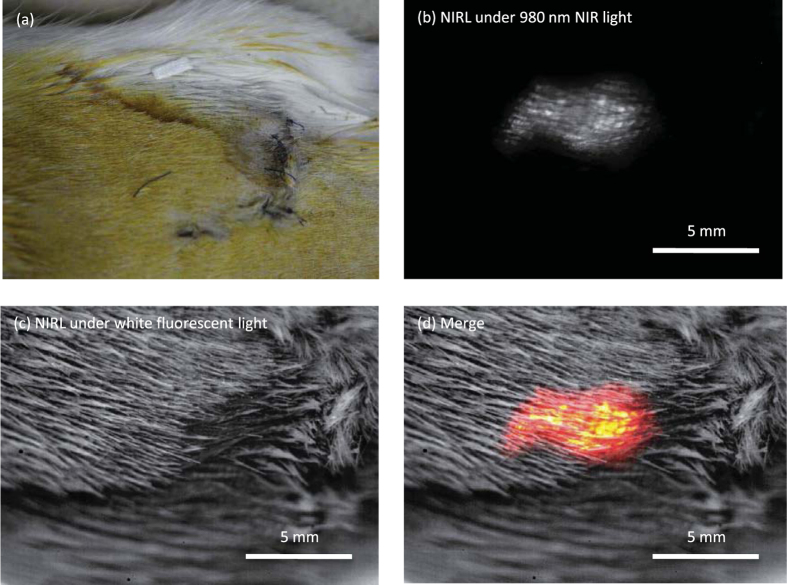
*In vivo* NIRL imaging of the cell sheet in a rat. (**a**) Photograph of the back of a rat. The cell sheet was implanted under the L-shaped stitch. *In vivo* NIRL images of Y_2_O_3_:Er, Yb NPs in the cell sheet transplanted onto the back of the rat under 980 nm NIR light irradiation (**b**). NIR reflection image of the back of the rat under white fluorescent lamp illumination (**c**). For NIRL imaging, the acquisition wavelength was 1525–1575 nm. The intensity of 980 nm NIR light was approximately 200 mW/cm^2^. Exposure time was 1 s. (**d**) Merged images of (**b**,**c**).

For *in vivo* deep tissue imaging, we used a cell sheet, which is a sheet-like cluster of cells. Cell sheets have been shown to be able to repair organs, e.g. the cornea and heart, in the field of regenerative medicine^20^,^21^. The cell sheet is approximately 1 cm in diameter and 50 μm in thickness. After introduction of the Y_2_O_3_:Er, Yb NPs to a cell sheet composed of C2C12 cells, the sheet was transplanted onto the back of a hairy rat. The location of the cell sheet was revealed by NIRL-based deep tissue observation. Figure 4(a) shows the image of the back of the hairy rat acquired with a colour CCD camera. The surface of the rat skin was covered with 2 mm long hair and did not reveal the internal condition of the body. However, when 980 nm NIR light was irradiated on the back of the rat, it penetrated the skin and hair of the rat, and consequently, the location of the cell sheet was observed. NIRL around 1530 nm emitted from the NPs was detected with a 2D InGaAs camera as shown in Fig. 4(b). The 980 nm excitation light was guided through an optical fibre, the emitted NIRL was detected through the lens unit of a single-lens reflex camera, and optical filters were used to cut off the laser light (Fig. S3). Figure 4(c) shows the image of the back of the rat where the cell sheet was implanted, acquired with the InGaAs camera under white fluorescent lamp illumination. Figure 4(d) shows merged images of the back of the rat acquired with the 2D InGaAs camera under the conditions of exposure to 980 nm NIR light and the white fluorescent lamp, confirming that the cell sheet adhered to the fascia of the rat.

**Figure 5:**
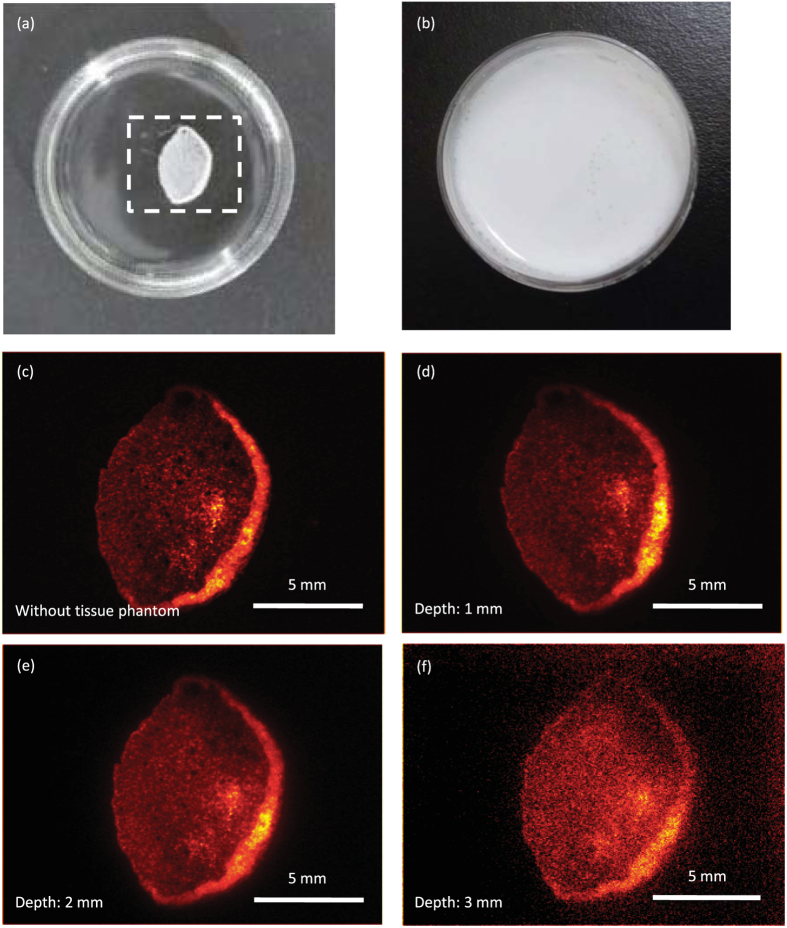
NIRL imaging of the cell sheet through the tissue phantom (2% IL). Photograph of the cell sheet containing Y_2_O_3_:Er, Yb NPs in a plastic dish (**a**). The white dotted-line square indicates observation area with the InGaAs camera. Photograph after pouring 1 mm 2% IL (**b**). NIRL images of the cell sheet without 2% IL (**c**), with 1 mm (**d**), 2 mm (**e**), and 3 mm (**f**) thick 2% IL. For NIRL imaging, the acquisition wavelength was 1525–1575 nm. The intensity of 980 nm NIR light was approximately 200 mW/cm^2^. Exposure time was 10 ms (**c**), 100 ms (**d**), 100 ms (**e**), and 1000 ms (**f**).

The implanted cell sheet was clearly observed even under the hairy rat skin of approximately 1 mm thickness. To determine the imaging depth limitation of the cell sheet with Y_2_O_3_:Er, Yb NPs, a turbid tissue phantom (2% Intralipid (IL)) that did not exhibit strong absorption was used. The cell sheet immersed in the phantom was observed with the same setup used for imaging of the rat (Fig. S4) while varying the thickness of the phantom from 1 mm to 3 mm. Figure 5(a,b) show the images of the cell sheet on a plastic dish and after pouring a 1-mm-thick phantom. Figure 5(c) is NIRL image of the cell sheet without the tissue phantom. The cell sheet was invisible when using visible light (Fig. 5(b)), but was clearly observed using 980 nm excitation and detection of 1530 nm NIRL from the Y_2_O_3_:Er, Yb NPs (Fig. 5(d,e)). Even with a 3-mm-thick tissue phantom, the shape and the folded part in the right edge of the cell sheet were detectable, though the image was blur (Fig. 5(f)). These results indicate that implanted tissues can be observed *in vivo* if the target is close to the skin, e.g. part of the liver, intestine, and so on.

### Extending the imaging depth of fluorescence microscopy with NIR excitation–NIR emission of Y_2_O_3_:Tm, Yb NPs

**Figure 6:**
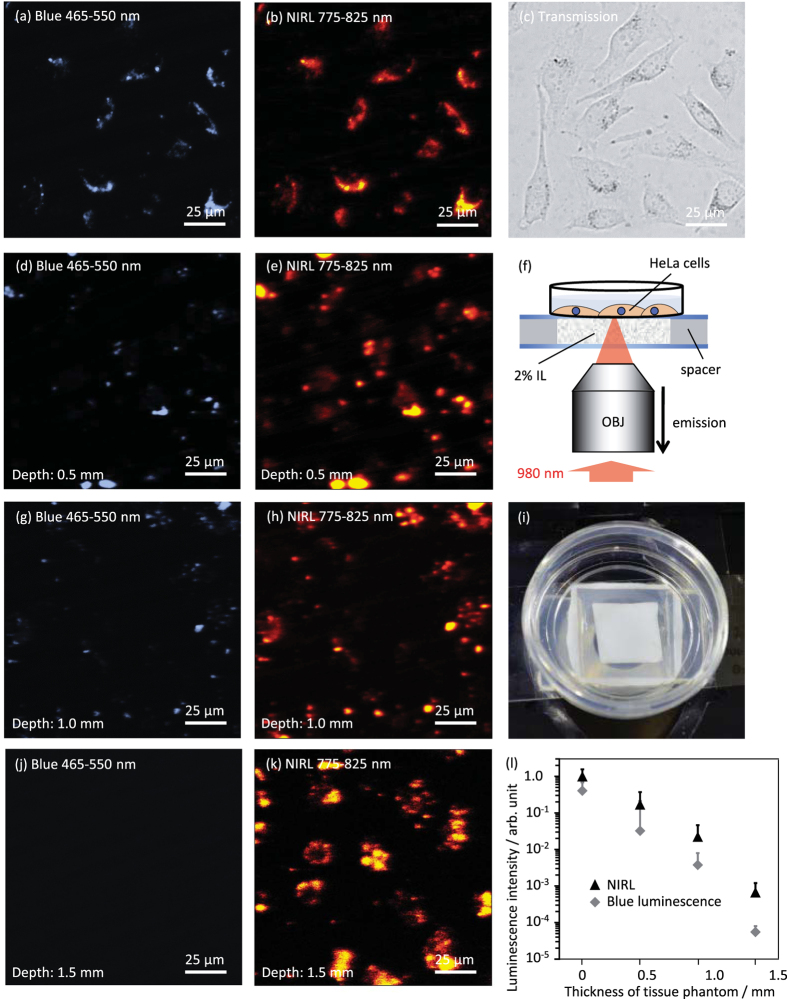
NIRL images of Y_2_O_3_:Tm, Yb NPs in HeLa cells. Luminescence images of cells at the same observation area observed without 2% IL (**a,b**), through 0.5 mm (**d,e**), 1.0 mm (**g,h**), and 1.5 mm (**j,k**) thick 2% IL. The bright-field image without 2% IL (**c).** Schematic of NIR imaging (**f**). Photograph of 2% IL (**i**). The acquisition wavelength was 465–550 nm in blue luminescence images and 775–825 nm in NIRL images. The intensity of 980 nm NIR CW laser light was 14.5 mW. Scanning speed was 61.44 μs/pixel. Luminescence intensity changes of Y_2_O_3_:Tm, Yb NPs with the thickness of the tissue phantom (**l**).

The penetrative ability of NIRL was compared with that of visible luminescence imaging. Figure 6 shows the images of HeLa cells observed through different thicknesses of the tissue phantom (2% IL). To mimic deep observation through the skin tissue, the 980 nm excitation light and emissions from the Y_2_O_3_:Tm, Yb NPs were made to pass through the tissue phantom (Fig. 6(f)). To confirm the observation depth of NIR excitation–NIR emission imaging, intensity changes of blue luminescence around 490 nm and NIRL around 810 nm from Y_2_O_3_:Tm, Yb NPs inside the cells were investigated. As shown in Fig. 6(a,b), both blue luminescence and NIRL from the Y_2_O_3_:Tm, Yb NPs enables the clear visualization of cell locations, as observed in the bright field image (Fig. 6(c)). The spatial resolution of the blue luminescent image was higher than that of the NIRL image, which is due to the higher-order non-linear excitation process of the blue luminescence^22^. However, blue luminescence signals decreased considerably with a visually turbid tissue phantom (Fig. 6(i)), while NIRL signals clearly indicated the location of the NPs inside the cells through 1.5 mm thickness of the tissue phantom (Fig. 6(d,e,g,h,j,k)). Figure 6(l) shows intensity changes of blue luminescence and NIRL images obtained for different thicknesses of the tissue phantom. Both the blue luminescence and NIRL intensities were calculated with correction of spectral sensitivity and amplification in a photomultiplier tube. While the average intensity of NIRL signals originating from the Y_2_O_3_:Tm, Yb NPs was double that of blue luminescent signals without a tissue phantom (Fig. 6(a,b)), the NIRL signals were 80 times higher than the blue luminescence signals when using the 1.5 mm tissue phantom (Fig. 6(j,k)). Thus, using the NIR wavelength region for excitation and emission allowed the imaging depth to be extended to the millimetre scale.

### Multicolour cellular CL imaging using Y_2_O_3_:Tm, Yb and Y_2_O_3_:Er, Yb NPs

**Figure 7:**
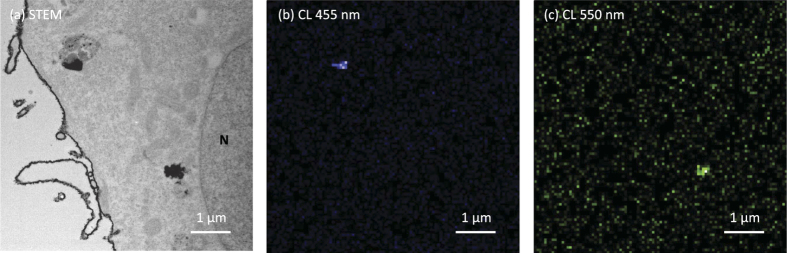
Cellular STEM image (**a**) and CL images (**b,c**) of Y_2_O_3_:Tm, Yb NPs and Y_2_O_3_:Er, Yb NPs. Excitation source: electron beam. Acquisition wavelength: 455 nm (**b**), 550 nm (**c**); Acceleration voltage of electron beam: 80 kV; Image size: 7 × 7 μm; Pixel size: 70 nm; Scanning speed: 500 ms/pixel. The nuclear region is marked with ‘N’ in the STEM image.

Locations of the two types of Y_2_O_3_ NPs in the HeLa cells were visualized using a scanning transmission electron microscopy (STEM)-CL system. Figure 7(a) shows the STEM image of ultra-thin sections of HeLa cells. Two NP clusters were located in the cytoplasm; however, the NP type was not recognized from the monochrome STEM image. In contrast, Fig. 7(b,c) show that in spectral CL images, a strong blue CL around 455 nm is emitted from the Y_2_O_3_:Tm, Yb NP cluster located at the top-left side, and the green CL around 550 nm is emitted from the Y_2_O_3_:Er, Yb NP cluster at the bottom-left. Thus, the locations of the Y_2_O_3_:Tm, Yb and Y_2_O_3_:Er, Yb NPs were visualized based on the CL colour. The advantage of STEM-CL imaging over super-resolution microscopy is that not only the target probe, but also cellular components can be observed with high spatial resolution^11^. Mitochondria are observable in the STEM image; in addition, some staining methods can be used to enhance specific cellular components such as actin, membrane, and cytoplasmic structures, which would help analysing cells further^23^.

## Discussion

We demonstrated a new correlative imaging technique with NIRL and CL using rare-earth doped NPs, Y_2_O_3_:Tm, Yb and Y_2_O_3_:Er, Yb NPs, which exhibited luminescence in different wavelength regions under both 980 nm NIR light and electron beam irradiation. Although various imaging probes have been designed for NIR imaging, including fluorescent proteins^24^,^25^, carbon nanotubes^26^, quantum dots^27^,^28^, and rare-earth complexes^29^, only a few candidates enable selection of the excitation and emission light in the NIR wavelength range. Some probes have also been studied for CL bioimaging, including fluorescent proteins^30^, inorganic fluorescent powders^31^, rare-earth complexes^32^, quantum dots^33^, and nanodiamonds^10^,^34^; in particular, quantum dots and nanodiamonds are promising imaging probes that combine different microscopy observations with photoluminescence under UV-visible light excitation and cathodoluminescence under accelerated electron excitation. The multimodalities of our rare-earth NPs will allow their use in multiscale correlative NIR excitation–NIR emission and CL imaging, which can be used to trace the region of interest at different scales, ranging from cellular dynamics to molecular distributions.

We also demonstrated multiscale bioimaging from *in vivo* to the subcellular level. In NIRL imaging, the NPs allowed deep-tissue imaging because highly penetrative NIR excitation and emission were used. Using this technique, the location of the cell sheet transplanted in the hairy rat was clearly visualized through the thickness (approximately 1 mm) of the skin and the hair. Thus, cell sheet imaging using the InGaAs camera has the potential to image up to a depth of 3 mm, which will be useful to trace implanted cell sheets in internal organs located close to an animal’s skin. The locations of the cells where the Y_2_O_3_:Tm, Yb NPs were introduced were clearly recognized even when using a 1.5 mm-thick tissue phantom. Usually, two-photon fluorescence microscopy enables observation at depths ranging from hundreds of micrometres to one millimeter^35^,^36^,^37^. We demonstrated that our NPs, when excited by continuous-wave NIR laser light, can be used for deeper observations. In addition to extending the depth limit of observation by NIRL imaging, the NPs realized high-spatial resolution CL imaging of subcellular components with multicolour. The types of NPs were clearly differentiated by CL colour. Thus, under both NIR light and electron beam irradiation, the NPs were recognized by their emission wavelength. The demonstrated results indicate the possibility of establishing a new correlative bioimaging technique, which will extend the applicability of the conventional CLEM technique to wide-scale and colour observation, including *in vivo*/deep observation. It is almost impossible to mimic cellular functions and interactions with surrounding tissue *in vitro* and hence *in vitro* studies cannot reflect the intracellular conditions; however, this technique has the potential to observe molecular dynamics *in vivo*, and subsequent CL imaging will allow single molecule-level analysis with cellular ultra-structures.

In recent years, several articles have been reported on the study of seamless correlative bioimaging techniques, such as live CLEM, correlative 3D-tomography, and atmospheric electron microscopy^2^,^4^,^38^,^39^. In addition to the NIRL/CL correlative imaging technique described here, the developed NPs are applicable to the other advanced CLEM techniques mentioned above. Our NPs have multimodalities, which allow their use in both light and electron microscopy imaging, including the conventional techniques and the new CLEM techniques described above.

## Methods

### Chemicals

Yttrium nitrate (Y (NO_3_)_3_ · 6H_2_O (99.9%)), thulium nitrate (Tm (NO_3_)_3_ · xH_2_O (99.9%)), and ytterbium nitrate (Yb (NO_3_)_3_ · xH_2_O (99.9%)) were purchased from Kojundo Chemical Laboratory Co., Ltd., Japan. Erbium nitrate (Er (NO_3_)_3_ · 5H_2_O (99.9%)) was purchased from Sigma-Aldrich Japan. Urea (Biochemistry grade) and ethanol (JIS Special Grade) were purchased from Wako Pure Chemical Industries, Ltd., Japan. All chemicals were used without further purification.

### Synthesis of Y_2_O_3_ NPs

NPs containing the rare-earth oxides—Y_2_O_3_:Tm 0.2 mol%, Yb 2 mol% and Y_2_O_3_:Er 3 mol%, Yb 20 mol% NPs—were synthesized according to the homogeneous precipitation method^19^,^40^. The synthetic procedure for Y_2_O_3_:Tm 0.2 mol%, Yb 2 mol% NPs is described here. Yttrium nitrate (273.84 μmol), thulium nitrate (0.56 μmol), and ytterbium nitrate (5.6 μmol) were dissolved in 40 mL of distilled water and the solution was poured into a 50 mL eggplant-shaped flask. Urea (0.457 mol) was added to the rare-earth nitrate solution and the solution was stirred vigorously until its temperature reached 20–25 °C. The flask was heated to 80 °C while stirring for 1 h to obtain precursors of NPs, which were subsequently separated from impurities and residual rare-earth nitrates by ultracentrifugation (25 krpm, 10 min, three times) (CP70MX, HITACHI). Then, the precursors were dispersed into ethanol, poured into an alumina melting pot, and dried at 110 °C. The dried precursors were calcined in an electric furnace (FT-101, Full Tech) at 1200 °C for 3 h to finally obtain Y_2_O_3_:Tm, Yb NPs.

### Characterization

Particles were observed with a transmission electron microscope (TEM, H-7650, Hitachi). Particle size was determined from TEM images using the ‘Analyze particles’ function of *ImageJ* software (National Institutes of Health). The concentrations of rare-earth elements in the NPs were calibrated with an inductively coupled plasma atomic emission spectroscope (ICP-AES, ICPS-8100, Shimadzu). Structural analyses of the NPs were conducted by using X-ray diffraction (XRD) with Cu Kα1 radiation (Rint2000, Rigaku). Near-infrared luminescent spectra of Y_2_O_3_ phosphors excited by a 980 nm NIR diode laser (IRM980TR-500, Laser Century) were measured with a spectrometer (NIRQUEST512, Oceanoptics) as shown in Fig. S1. Cathodoluminescent spectra and images of the NPs were measured with a spectrometer (TRIAX-320, Horiba-Jobin Yvon) and a cooled CCD camera (CCD-1024 × 256-4, Horiba-Jobin Yvon) placed in a CL measurement unit (Horiba) attached to an FE-SEM instrument (JSM-6500F, JEOL) as shown in Fig. S2.

### Correlative NIRL and CL imaging

NP locations were observed under 980 nm NIR light and electron beam irradiation. Each type of NP was dispersed on a 50 nm-thick silicon nitride membrane grid (SN100-A50Q33, SPI Supplies). Bright-field and NIRL images were obtained with a modified laser scanning microscope (C1, Nikon) equipped with a 980 nm NIR diode laser and a photomultiplier tube (H7844 or H10330B-75, Hamamatsu) as shown in Fig. S3. NIRL images were constructed by raster scanning using a galvano mirror. After NIRL imaging, CL images were obtained with the FE-SEM-CL system described in our previous study^17^. The emitted CL was directed to a spectrometer (TRIAX-320, Horiba-Jobin Yvon) and CL images were recorded with a photomultiplier tube-type detector (PMT, R943-02, Hamamatsu).

### *In vivo* NIRL imaging of transplanted cell sheet

All the animal experimental protocols in this study were approved by the Committee for Animal Experiments, Graduate School of Engineering Science, Osaka University (approval: ES 26-4-0) and the experiments were carried out in accordance with the Regulations on Animal Experimentation at Osaka University. Transplantation of the cell sheet and NIRL imaging were performed on a rat under anaesthesia. Mouse skeletal myoblast cells (C2C12) were condensed to mono-layered sheet shape and cultured on a thermo-responsive dish at 37 °C for three days. The Y_2_O_3_:Er, Yb NPs were dispersed in Dulbecco’s modified medium (DMEM; Sigma-Aldrich) and introduced into the cell sheet through the endocytosis reaction for 1 day. After detachment from the thermo-responsive dish at 25 °C, the cell sheet was transplanted onto the fascia of the back of a nine-week aged rat (LEW/CrlCrlj)^41^,^42^. The hair on the back was trimmed to 2 mm length. The location of the transplanted cell sheet in the hairy rat was observed under 980 nm NIR light. The cell sheet on the back of the rat was exposed to light with an intensity of 200 mW/cm^2^ emitted from a 980 nm NIR diode laser (RLTMDL-980-2W-5, Roithner Lasertechnik) using an optical fibre (Fig. S4). The emitted NIRL was gathered by a lens unit for single lens reflex camera (VF50095M, SPACECOM, 50 mm, F 0.95) and filtered with three long-pass filters (High Performance Long pass Filter 1100 nm 25 mm, OD4, Edmund Optics) and two band-pass filters (Hard Coated Band pass Filter 1550 nm 25 mm, OD4, Edmund Optics). NIRL images were obtained with a 2D InGaAs camera (NIRvana:640, Princeton Instruments) with an exposure time of 1 s per image.

### Cellular NIRL imaging using Y_2_O_3_:Tm, Yb NPs

HeLa cells (Health Science Research Resources Bank, Osaka, Japan) were cultured in the DMEM with 10% foetal bovine serum and 1% penicillin streptomycin at 37 °C in 5% CO_2_. Before being introduced into cells, the Y_2_O_3_:Tm, Yb NPs were dispersed in distilled water and were sterilized by exposure to UV light for 24 h, following which they were dispersed in the culture medium. Then, the cells were cultured for 24 h, and the NPs were introduced into the cells by an endocytosis reaction. Subsequently, the cells were rinsed with phosphate buffered saline (PBS (−), Wako) three times and fixed with 4% paraformaldehyde in PBS (−). Subsequently, the cells were observed with the modified laser scanning microscope shown in Fig. S3. The cells were exposed to 980 nm NIR light through 0–1.5 mm-thick 2% IL, which has a scattering coefficient similar to that of the human skin^43^,^44^. Visible blue luminescence and NIRL from the NPs were also detected through the tissue phantom. After NIRL imaging with a band-pass filter (Hard Coated Bandpass Filter 800 nm 25 mm, OD4, Edmund Optics), the filter was changed for acquisition of blue luminescence (FF01-510/84-25, Semrock). The same photomultiplier tube (H7844, Hamamatsu) was employed to compare the penetrability of visible luminescence and NIRL through tissue. The intensity of luminescent signals from the NPs inside cells was calculated from the luminescent images with correction of the spectral sensitivity and amplification in a photomultiplier tube.

### Cellular CL imaging using Y_2_O_3_:Tm, Yb NPs and Y_2_O_3_:Er, Yb NPs

The Y_2_O_3_:Tm, Yb NPs and Y_2_O_3_:Er, Yb NPs were introduced into the HeLa cells in the same manner as described above. After 24 h from the introduction of the NPs, the cells were fixed with 2.4% glutaraldehyde in PBS (−) and then treated with the standard process to observe biological specimens with TEM. The details of the sample preparation process were described in ref. 45. With the treated cells embedded in epoxy resin, thin sectioning of the cells was conducted and the sections of cells were placed on 50-nm-thick silicon nitride membrane grids (NG06-071N, Alliance Biosystems, Japan). CL images of the cells were obtained by using an STEM (JSM-2100F, JEOL)-CL system. The emitted CL was directed to a spectrometer with a parabolic mirror. Spectral CL images were recorded by using an electron multiplying CCD detector (Andor, DU920p-BU)^11^,^46^,^47^.

## Additional Information

**How to cite this article**: Fukushima, S. *et al.* Correlative near-infrared light and cathodoluminescence microscopy using Y_2_O_3_:Ln, Yb (Ln = Tm, Er) nanophosphors for multi-scale, multicolour bioimaging. *Sci. Rep.* **6**, 25950; doi: 10.1038/srep25950 (2016).

## Supplementary Material

Supplementary Information
