# Supplementary material for: Correlative near-infrared light and cathodoluminescence microscopy using Y2O3:Ln, Yb (Ln = Tm, Er) nanophosphors for multiscale, multicolour bioimaging
Source: Sci Rep. 2016 May 17;6:25950. doi: 10.1038/srep25950 (PMC4869039; doi:10.1038/srep25950)
Supplement: Supplementary Information [file srep25950-s1.pdf]

Electronic supplementary information

Title: Correlative near-infrared light and cathodoluminescence microscopy using Y_2O_3:Ln, Yb (Ln = Tm, Er) nanophosphors for multiscale, multicolour bioimaging

Authors

S. Fukushima[1], T. Furukawa[2], *H. Niioka[1], M. Ichimiya[1,3], T. Sannomiya[4], N. Tanaka[5], D. Onoshima[6,7], H. Yukawa[7,8], Y. Baba[6,7,8,9], M. Ashida[1], J. Miyake[1], T. Araki[1], and M. Hashimoto[1]

[1]Graduate School of Engineering Science, Osaka University 1-3 Machikaneyama, Toyonaka, Osaka 560-8531, Japan.

[2]Institute for NanoScience Design, Osaka University 1-3 Machikaneyama, Toyonaka, Osaka 560-8531, Japan.

[3]School of Engineering, The University of Shiga Prefecture 2500 Hassaka-cho, Hikone, Shiga 522-8533, Japan.

[4]Department of Innovative and Engineered Materials, Tokyo Institute of Technology, 4259 Nagatsuta, Yokohama, Kanagawa 226-8503, Japan.

[5]Quantitative Biology Center, RIKEN, 6-2-3, Furuedai, Suita, Osaka 565-0874, Japan.

[6]Institute of Innovation for Future Society, Nagoya University, Furo-cho, Chikusa-ku, Nagoya 464-8603, Japan.

[7] ImPACT Research Center for Advanced Nanobiodevices, Furo-cho, Chikusa-ku, Nagoya 464-8603, Japan.

[8] Graduate School of Engineering, Nagoya University, Furo-cho, Chikusa-ku, Nagoya 464-8603, Japan.

[9]Health Research Institute, National Institute of Advanced Industrial Science and Technology (AIST), 2217-14, Hayashi-cho, Takamatsu 761-0395, Japan.

* Corresponding author. Tel.: +81-6-6850-6550. E-mail address: niioka@bpe.es.osaka-u.ac.jp.

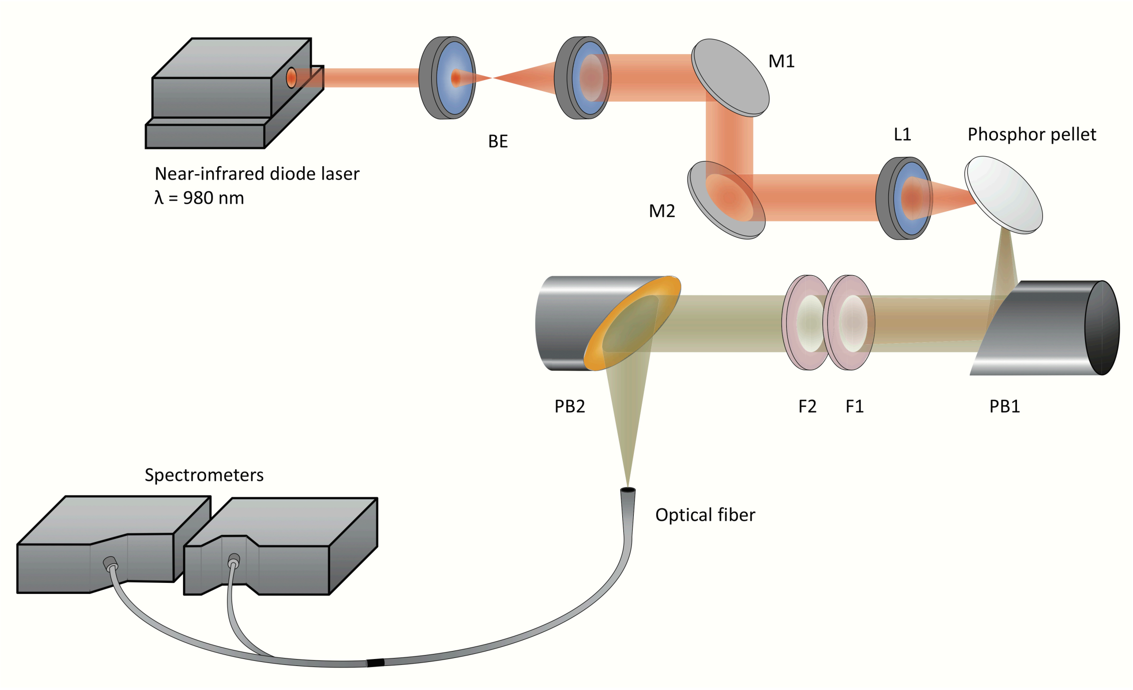

Fig. S1: Optical setup for the acquisition of luminescence from Y_2O_3 phosphor pellets. Setup included a near-infrared diode laser (IRM980TR-500, Laser Century); a pair of achromatic lenses (AC-254-075-C, Thorlabs) as a beam expander, BE; and silver-coated mirrors M1 and M2. Near-infrared laser light was focused onto phosphor pellet by an achromatic lens (AC-254-075-C, Thorlabs; L1) and filtered by a short-pass filter (BlightLine, fluorescence filter 950/SP, Semrock; F1) and a long-pass filter (RazorEdge LongPass 980, Semrock; F2). Luminescence from the phosphor pellet was collected by silver-coated parabolic mirror (MPD254254-90-P01, Thorlabs; PB1, PB2) and was led to a spectrometer (USB4F10023, Oceanoptics, NIRQUEST, NQ51A0577, Oceanoptics) through a two-branched optical fibre (CUSTOM-BIF-6174546, 1000 um VIS/NIR, Oceanoptics). The obtained spectral intensity was calibrated by using a light source device (HL2000, Oceanoptics).

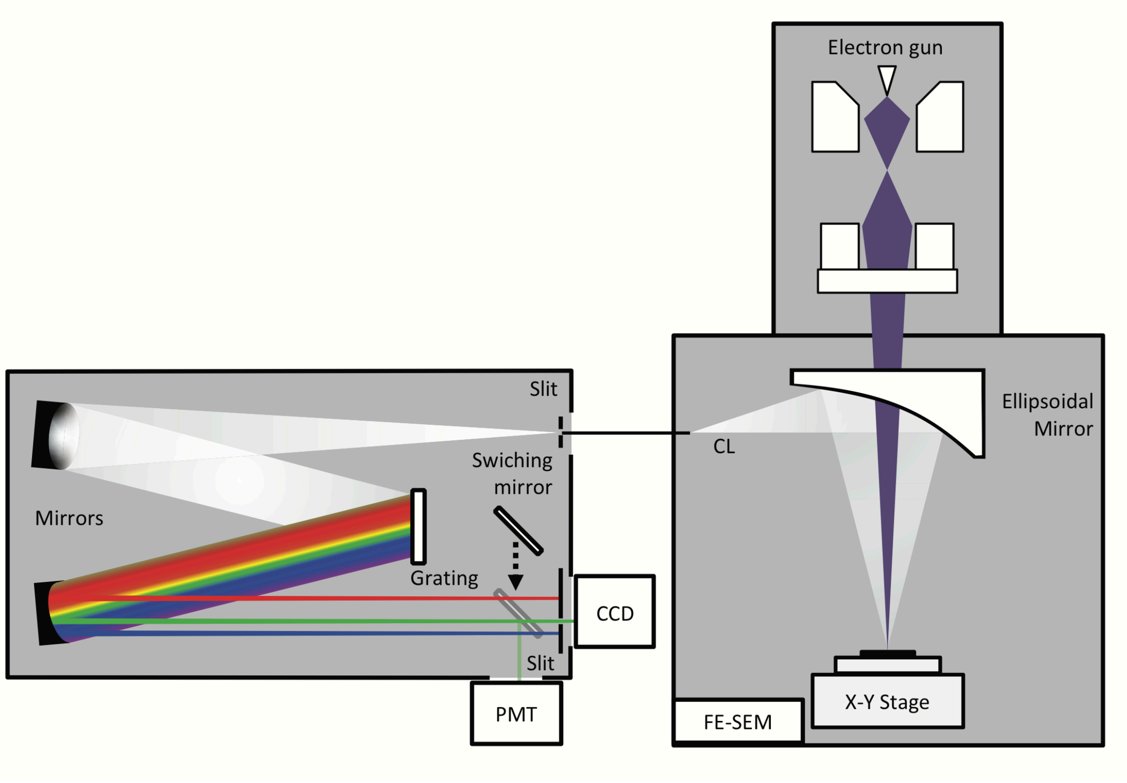

Fig. S2: Experimental setup for SEM-CL imaging. Accelerated electrons excite specimens, and secondary electrons and CL are emitted. Ellipsoidal mirror leads CL to spectrometer (TRIAX-320, Horiba-Jobin Yvon) through a quartz optical fibre. CL spectrum is obtained by a cooled CCD camera (CCD-1024×256-4, Horiba-Jobin Yvon). CL image is constructed with photomultiplier tube (R943-02, Hamamatsu).

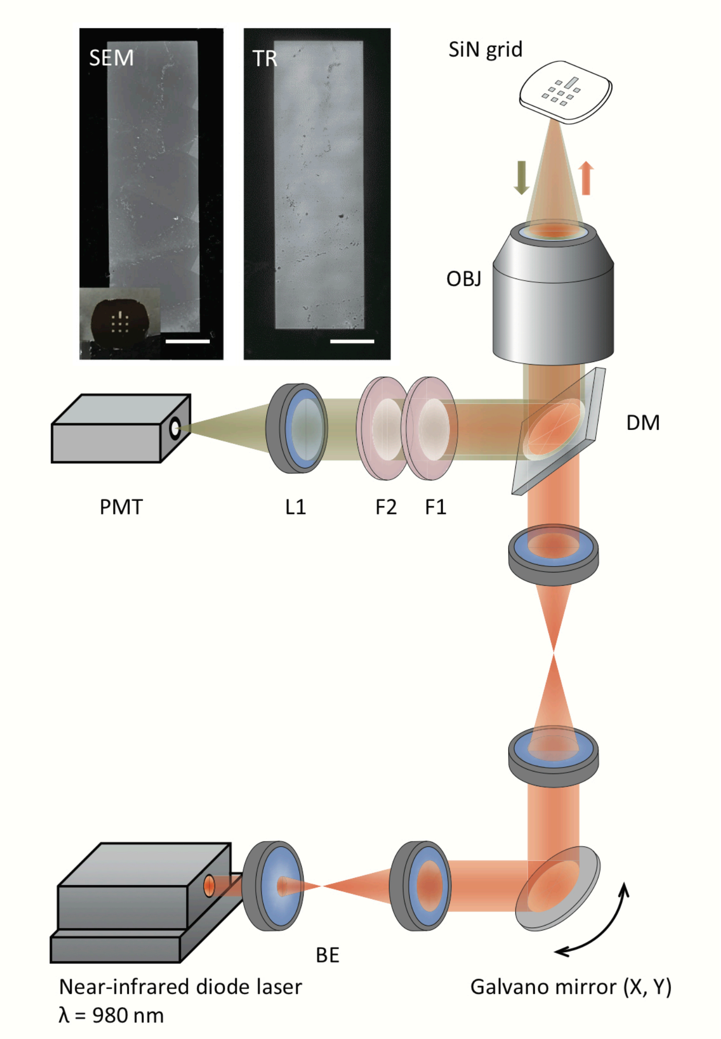

Fig. S3: Optical setup for NIR imaging, including a near-infrared diode laser (MDL-III-980/1～2000mW, CNI) and a pair of achromatic lenses (AC-254-075-C, Thorlabs) as a beam expander, BE. This setup was constructed with commercial laser scanning microscope (C1, Nikon).

For acquisition of near-infrared luminescence from Y_2O_3:Tm, Yb NPs, an objective lens (LCPlan N 20x, NA 0.45, Olympus), OBJ; a dichroic long-pass filter (FF87501-25x36, Semrock), DM; a short-pass filter (FF01-950/SP-25, Semrock), F1; a band-pass filter (Hard Coated Bandpass Filter 800 nm 25 mm, OD4, Edmund Optics), F2); and a photomultiplier tube (Hamamatsu, H7844, Hamamatsu), PMT, were used. Visible luminescence from Y_2O_3:Tm, Yb NPs was acquired with the same optical setup except for the objective lens (LR Plan NIR 20x, NA 0.4) and the band-pass filter (FF01-510/84/25, Semrock).

For acquisition of NIRL from Y_2O_3:Er, Yb NPs, an objective lens (M Plan Apo NIR 20x, NA 0.4, Mitutoyo), OBJ; a dichroic short-pass filter (TS dichroic short-pass 1200 nm, Edmund Optics), DM; a long-pass filter (High Performance Longpass Filter 1100 nm 25 mm, OD4, Edmund Optics), F1; a band-pass filter (Hard Coated Bandpass Filter 1550 nm 25 mm, OD4, Edmund Optics), F2; and a near-infrared photomultiplier tube (H10330B-75, Hamamatsu), PMT, were used. Insets: SEM and transmission (TR) images of SiN membrane grid. Scale bar: 50 µm.

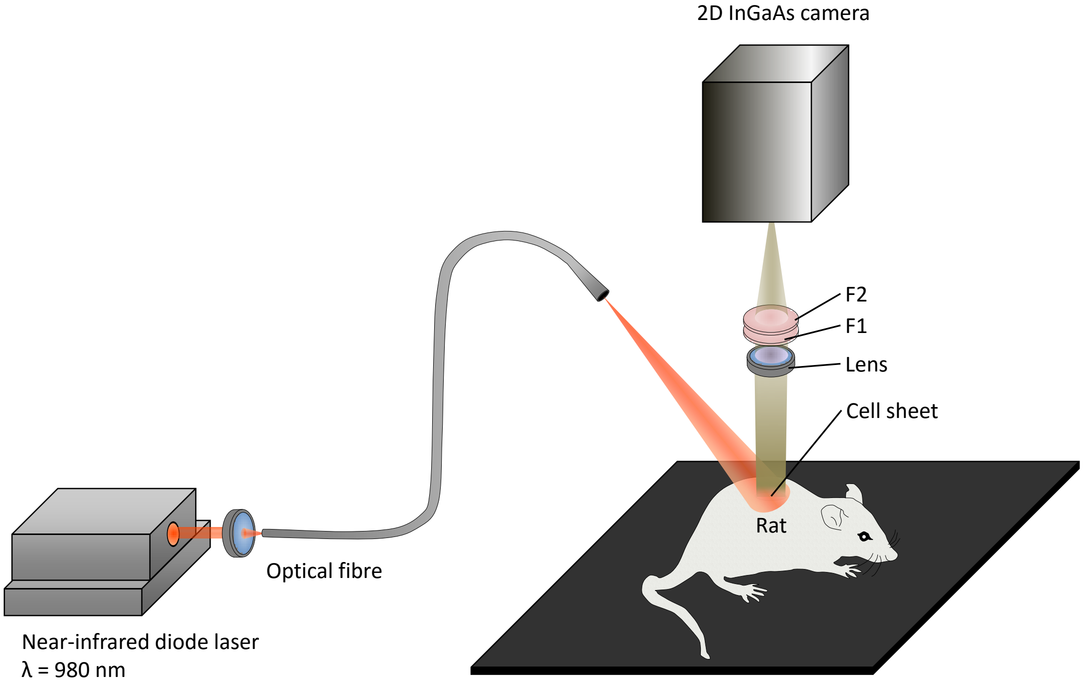

Fig. S4: Optical setup for the *in* vivo NIRL deep tissue observation of cell sheet transplanted in the back of a hairy mouse and *in vitro* cell sheet imaging with tissue phantom (2% intralipid). An inbred mouse was illuminated by 980 nm near-infrared laser light under anesthesia. Setup included a near-infrared diode laser (IRM980TR-500, Laser Century); optical fibre (CUSTOM-BIF-6174546, 1000 um VIS/NIR, Oceanoptics), Lens (VF50095M, SPACECOM, 50 mm, F 0.95), a long-pass filter (High Performance Longpass Filter 1100 nm 25 mm, OD4, Edmund Optics), F1; a band-pass filter (Hard Coated Bandpass Filter 1550 nm 25 mm, OD4, Edmund Optics), F2; and a 2D InGaAs CCD (NIRvana:640, Princeton Instruments).
